# Percutaneous Ultrasound-Guided Carpal Tunnel Release: Study Upon Clinical Efficacy and Safety

**DOI:** 10.1007/s00270-016-1545-5

**Published:** 2016-12-27

**Authors:** David Petrover, Jonathan Silvera, Thierry De Baere, Marie Vigan, Antoine Hakimé

**Affiliations:** 1 Department of Interventional Radiology, Imagerie Médicale Paris Centre Bachaumont-clinique Blomet RamsayGDS, 135 bis rue Blomet, 75015 Paris, France; 2 Gustave Roussy Institute, Villejuif, France; 3 Association pour la recherche en chirurgie de l’épaule et du coude, clinique Drouot, 20 rue Laffitte, 75009 Paris, France

**Keywords:** Carpal tunnel syndrome, Magnetic resonance imaging, Interventional ultrasound, Minimally invasive surgical procedures, Surveys and questionnaires

## Abstract

**Objectives:**

To evaluate the feasibility and 6 months clinical result of sectioning of the transverse carpal ligament (TCL) and median nerve decompression after ultra-minimally invasive, ultrasound-guided percutaneous carpal tunnel release (PCTR) surgery.

**Methods:**

Consecutive patients with carpal tunnel syndrome were enrolled in this descriptive, open-label study. The procedure was performed in the interventional radiology room. Magnetic resonance imaging was performed at baseline and 1 month. The Boston Carpal Tunnel Questionnaire was administered at baseline, 1, and 6 months.

**Results:**

129 patients were enrolled. Significant decreases in mean symptom severity scores (3.3 ± 0.7 at baseline, 1.7 ± 0.4 at Month 1, 1.3 ± 0.3 at Month 6) and mean functional status scores (2.6 ± 1.1 at baseline, 1.6 ± 0.4 at Month 1, 1.3 ± 0.5 at Month 6) were noted. Magnetic resonance imaging showed a complete section of all TCL and nerve decompression in 100% of patients. No complications were identified.

**Conclusions:**

Ultrasound-guided PCTR was used successfully to section the TCL, decompress the median nerve, and reduce self-reported symptoms.

## Introduction

Carpal tunnel syndrome, which is a common neuropathy, is caused by the transverse carpal ligament (TCL) compressing the median nerve at the base of the palm. Most often, when nonsurgical methods, such as rest, splinting, physical therapy, and corticosteroid injections, do not alleviate symptoms sufficiently, a surgical release of the median nerve is achieved by sectioning the TCL.

Open carpal tunnel releases (OCTR) have been performed successfully for many years [1]. These procedures are, however, associated with 60–80 mm scars, lengthy recovery periods (25 days), and a complication rate of ~1% [2]. Endoscopic techniques (ECTR) have been developed as a less invasive alternative. Although endoscopic procedures reduce scarring to 10 mm, this technique can be challenging because the initial placement of the trocar is blind and during the procedure, vision is limited by the narrow range of the endoscope. In fact, meta-analyses have underscored the impact of low visibility by showing that risk of transient nerve damage is higher with ECTR than with OCTR [3, 4]. Overall improvement and reoperation rates are, however, similar; and other post-operative variables, such as recovery time, strength during the early post-operative period, and wound problems (scar tenderness, infection, hypertrophic scarring), are significantly better with ECTR than with OCTR [3, 4].

Recent developments in sonography now allow us to demarcate superficial soft tissues and to identify very small anatomic and pathologic details. In the context of carpal tunnel release surgery, a technique that incorporates the careful delineation of the thenar motor branch of the median nerve, for instance, may help reduce the risk of complications due to nerve damage [5]. To this effect, ultrasound-guided percutaneous carpal tunnel release (PCTR) has been developed. This technique makes use of the detailed anatomical information that can be gathered from continuous ultrasound monitoring and combines it with the advantages of minimally invasive percutaneous surgery [6–11].

Studies on cadavers have shown that the transverse carpal ligament can be transected successfully using PCTR [7, 8, 12, 13]; and clinical studies have shown that PCTR is effective and well tolerated [6, 9, 11, 14]. None of these clinical studies, however, have documented outcomes using magnetic resonance imaging (MRI), which is the gold standard for confirming complete sectioning of transverse carpal ligament and surgical decompression of the median nerve [15–17]. Furthermore, in all these studies the operator was not an interventional musculoskeletal radiologist, and it is unclear how many years of experience the operator had with hand ultrasound and percutaneous ultrasound-guided procedures. The primary objective of this prospective clinical study is to assess the feasibility and safety of PCTR when it was performed by an interventional radiologist and to use MRI to document it. The secondary objective was to examine efficacy.

## Methods

In this prospective, open-label PCTR study, consecutive patients were enrolled if they had been referred for carpal tunnel surgery, had carpal tunnel syndrome symptoms for more than 6 months, had a confirmation of diagnosis by electromyogram, and had failed medical treatment. Patients with a history of carpal tunnel release surgery were excluded. Informed consent was obtained from all individuals included in the study. Institutional Review Board approval was obtained for this study.

**Fig. 1:**
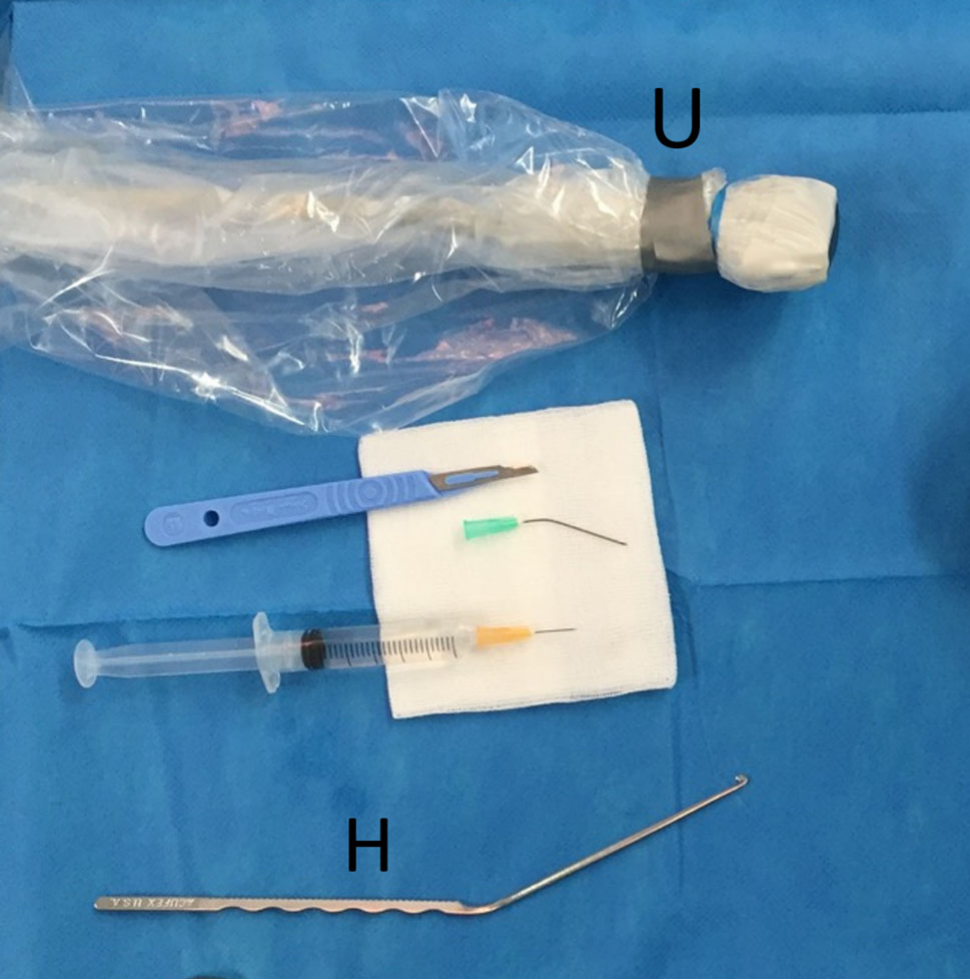
Standard table for PCTR. *H* Hook knife. *U* Ultrasound probe in a sterile covering

Sonographic evaluations and surgery were performed by one interventional radiologist with 12 years of experience in musculoskeletal sonography. All PCTR were ambulatory and were performed in the interventional radiology room under local anesthesia (Fig. 1). Just prior to starting the procedure, the median nerve, the ulnar nerve, the vascular palmar arch, and the transverse carpal ligament were mapped using a Hitachi Noblus ultrasound scanner (Hitachi Medical Systems Europe, Zug, Switzerland) with an 18 MHz probe. Emphasis was placed on the charting of the motor and sensitive branches of the median nerve and on the identification anatomic variations. Results of this sonographic evaluation were used to determine the area of incision and a safe release path [6, 8, 11].

The retrograde division of the transverse carpal ligament was then carried out using the 3-step UGS (Ultrasound-Guided Surgery) with a distal antebrachial approach.

**Fig. 2:**
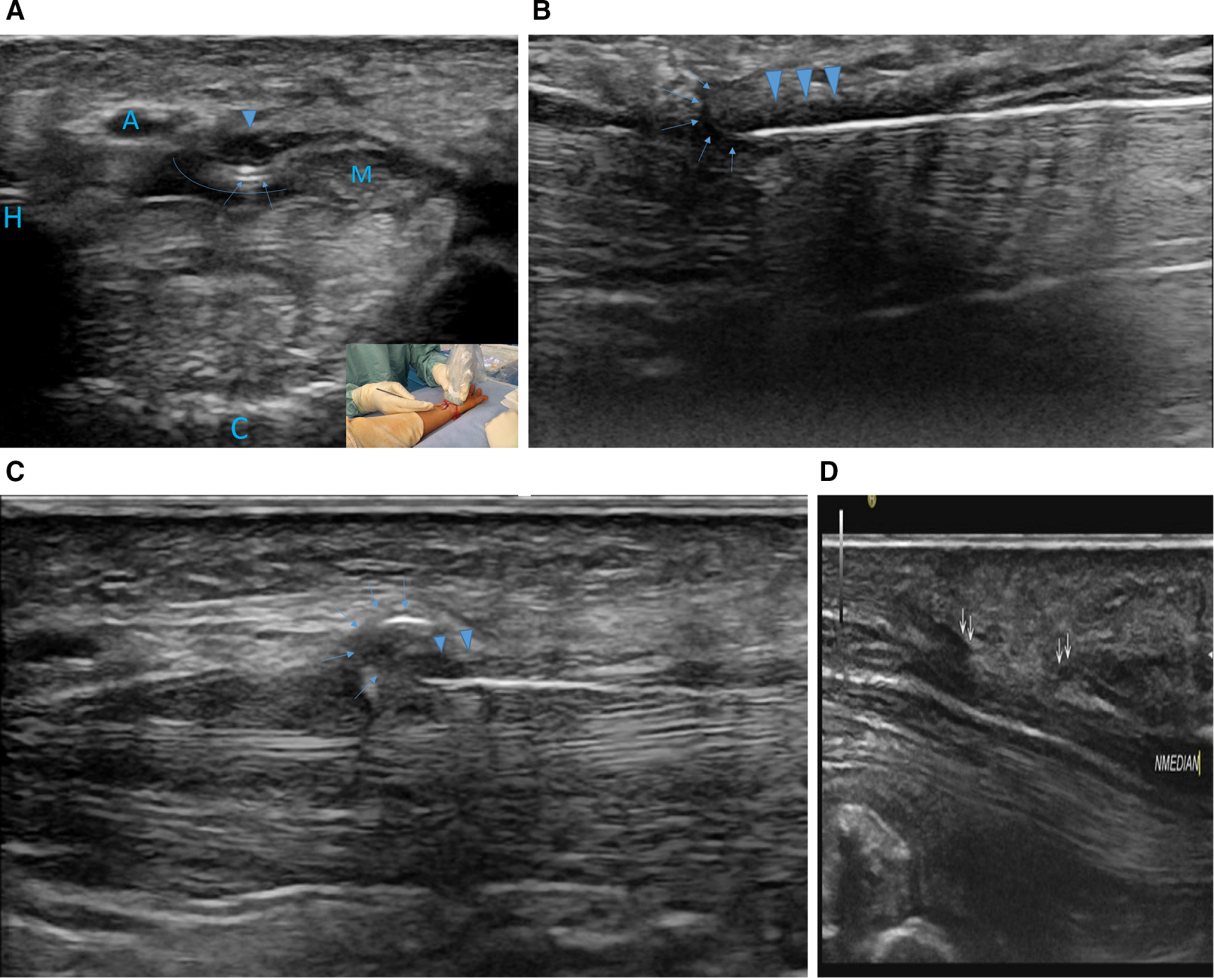
Ultrasound monitoring of the ultra-minimally invasive carpal tunnel release. **A** axial view: hook knife position (*arrow*) between the median nerve (M) and ulnar artery (A) and below the transverse carpal ligament (*arrowhead*); the *curved line* represent the space expand with local anesthetic between the carpal tunnel ligament, the flexor tendons, the hook of the hamatum and the median nerve. **B** *Longitudinal view* positioning of the hook knife (*arrow*) at the distal cutting point below the TCL (*arrowhead*). **C** *longitudinal view* pulling back on the hook knife (*arrow*) while applying volar pressure for retrograde releasing of the TCL (*arrowhead*). **D** ultrasound control of the TCL release. *Double arrow* represents the free edges of the TCL that have been cut releasing the median nerve (NMedian). Bone landmarks; *H* is the hook of the hamatum, *C* is the capitatum

During step 1, local anesthesia was administered using a 26 gage needle at the wrist crease, and using a 22 gage needle inside the carpal tunnel just below the TCL centered above the capitatum and lunatum bone. 3 cc of local anesthetic was able to fill and expand the space between the median nerve, the hook of the hamatum, the TCL and the flexor tendon called the longitudinal safe zone. During step 2, a point of entry at the proximal wrist crease was pierced through the deepest fibrous layer using a scalpel 16. During step 3, an Acufex 3.0-mm hook knife (010600; Smith & Nephew PLC, London, England) was then advanced through the prepared longitudinal safe zone to the distal TCL. The hook knife was than rotated to point up to ensure that the blade was perpendicular to and hooked onto the TCL than pulled to perform the retrograde section. All steps including anesthesia were performed under ultrasonographic guidance (Fig. 2). Follow-up appointments took place 4 days, 1, and 6 months after surgery.

### Evaluations

Surgical procedure criteria were duration of the PCTR (not including preliminary ultrasound mapping) and total time in the procedure room.

The main clinical evaluation was the Boston Carpal Tunnel Questionnaire (BCTQ), which was administered prior to the procedure (baseline), 1 month after the procedure (Month 1), and at the end of the study (Month 6). The BCTQ is a 19-question, self-administered, patient questionnaire that evaluates severity of symptoms (11 questions) and functional status (8 questions) using a 5-point scale (1 = best score and 5 = worst score) [18, 19]. Additional clinical variables included evaluation of the scar and post-operative complications at Day 4, Month 1, and Month 6.

Magnetic resonance imaging was performed prior to surgery and 1 month after surgery using T1-weighted and T2-weighted fat saturation sequences (Magnetom^®^ Essenza 1.5T [Siemens, Erlangen, Germany]). The main imaging variable, the degree of sectioning of the TCL (absent, partial, or complete) and the size of the discrete gap in the TCL were determined from T2 axial image slices. T2 axial images were also used to evaluate the median nerve. The cross-sectional area of the median nerve was determined at the level of the hook of the hamate where the nerves tended to be most compressed. Decompression of the nerve was scored based on its position compared to the line joining the hook of hamate to the ridge of the trapezium. Position was defined as deep if the nerve was above the line; intermediary if the nerve crossed the line; superficial if nerve was under the line [17]. Decompression was considered successful if the nerve location moved from a deep position to a more superficial position after PCTR. Potential complications were also assessed by MRI. Images were read independently by the operating radiologist (DP) and by a radiologist (JS) who was blinded to clinical outcome. Any discrepancies were resolved by consensus.

### Statistical Analysis

Descriptive statistics were used to describe results. Categorical variables are presented as percentages. Continuous variables are presented as means and standard deviations (SD). McNemar tests were used to compare categorical variables. Student’s tests and Student’s t-tests for paired samples were used to compare continuous variables. The percentage of patients that showed a change in nerve position before and after PCTR and 95% confidence intervals were calculated. *P*-values were assessed at the 0.05 level. Statistical analyses were performed using SAS^®^ version 9.4 (SAS Institute Inc., Cary, NC, USA).

## Results

**Fig. 3:**
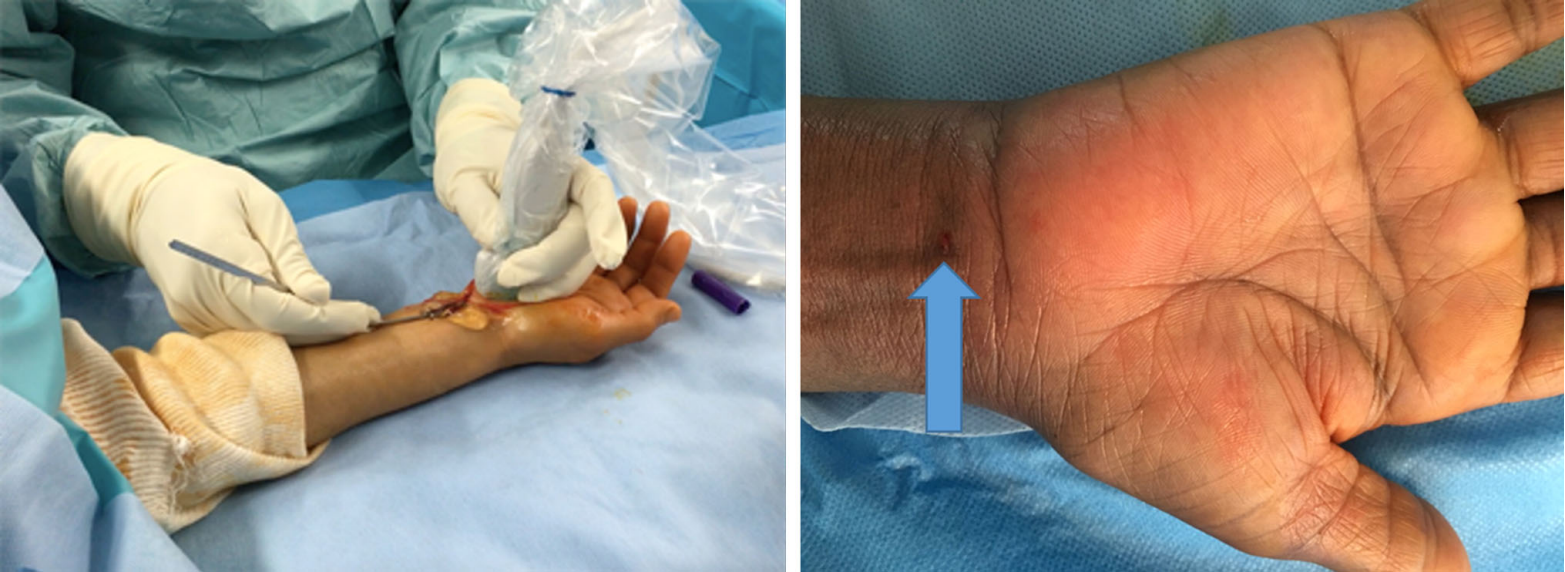
Surgical scar for ultra-minimally invasive carpal tunnel release. *Left* skin incision with the hook knife introduced percutaneously at the first available antebrachial skin crease in the left hand. *Right*, skin scar (*arrow*) just after

129 patients meeting study criteria were enrolled between January 2015 and June 2016. Baseline, Day 4, and Month 1 data were collected for all patients. Month 6 follow-up data, which were collected after a mean of 7.5 ± 2.8 months, were available for all patients. At baseline, mean age was 61.5 ± 13.3 years. Most patients were female (69.8%). Sixty-six patients (51.2%) needed surgery on their right hand and sixty-three (48.8%) on their left. Fifteen patients (11.6%) had a bifid median nerve. The procedure lasted a mean of 5.8 ± 2.4 min. Mean time in the procedure room was 23.2 ± 4.8 min. Scar length ranged from 2.0 to 5.0 mm (Fig. 3).

**Fig. 4:**
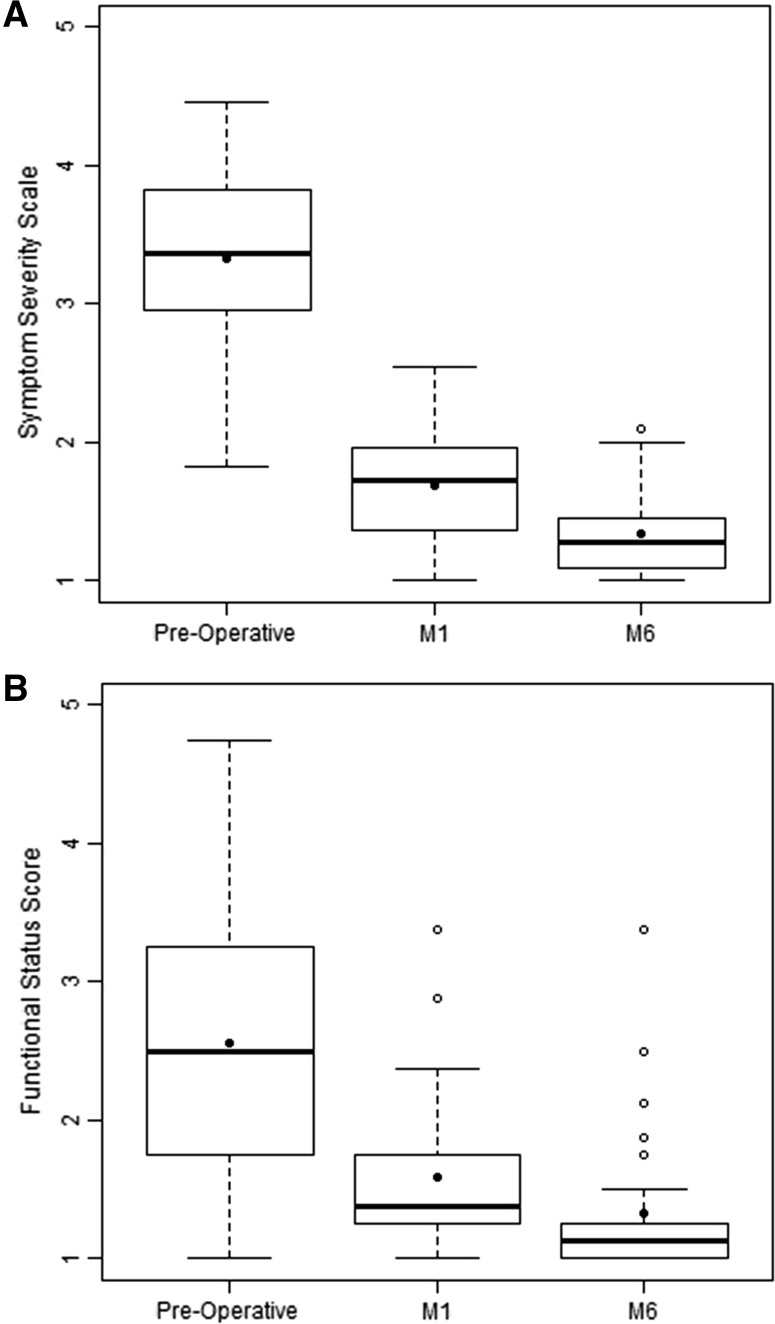
Boston Carpal Tunnel Questionnaire symptom and functional domain scores before and after minimally invasive ultrasound-guided percutaneous carpal tunnel release surgery. The Boston Carpal Tunnel Questionnaire (BCTQ) was administered prior to the procedure (pre-operative/baseline; *N* = 129), 1 month after the procedure (M1; *N* = 129), and after 6 months of follow-up (M6; *N* = 129). The BCTQ is a 19-question, self-administered patient questionnaire that uses a 5-point scale (1 = best score and 5 = worst score). **A** Symptom severity score (11 questions) and **B** Functional status score (8 questions). *Bolded horizontal line* represents the median. *Black full circle* represents the mean

No complications were reported at Day 4, Month 1, or Month 6. At 6 months, 12 patients (9.3%) reported minimal paresthesia. The BCTQ symptom severity score improved in all patients from a mean of 3.3 ± 0.7 at baseline to 1.7 ± 0.4 at Month 1 (Fig. 4A). The BCTQ functional status score improved from a mean of 2.6 ± 1.1 at baseline to 1.6 ± 0.4 at Month 1 (Fig. 4B). Changes from baseline to Month 1 were significant (*p* < 0.0001 for both domains). At Month 6, mean symptom severity score was 1.3 ± 0.3 and mean functional status score was 1.3 ± 0.5. Changes from the 1-month time point to the 6-month time point were significant for both domains (*p* < 0.0001 for symptom severity and *p* = 0.0004 for functional status).

Results of the MRI showed that a complete section of the TCL along its length was achieved in 129 patients (100%). The gap in the TCL measured a mean 5.1 ± 1.5 mm. At the level of the hamate bone, where the nerves tended to be most compressed at baseline, mean nerve cross-sectional surface area increased from 8.9 ± 3.3 mm^2^ at baseline to 13.5 ± 3.7 mm^2^ at Month 1 (*p* < 0.0001).

**Fig. 5:**
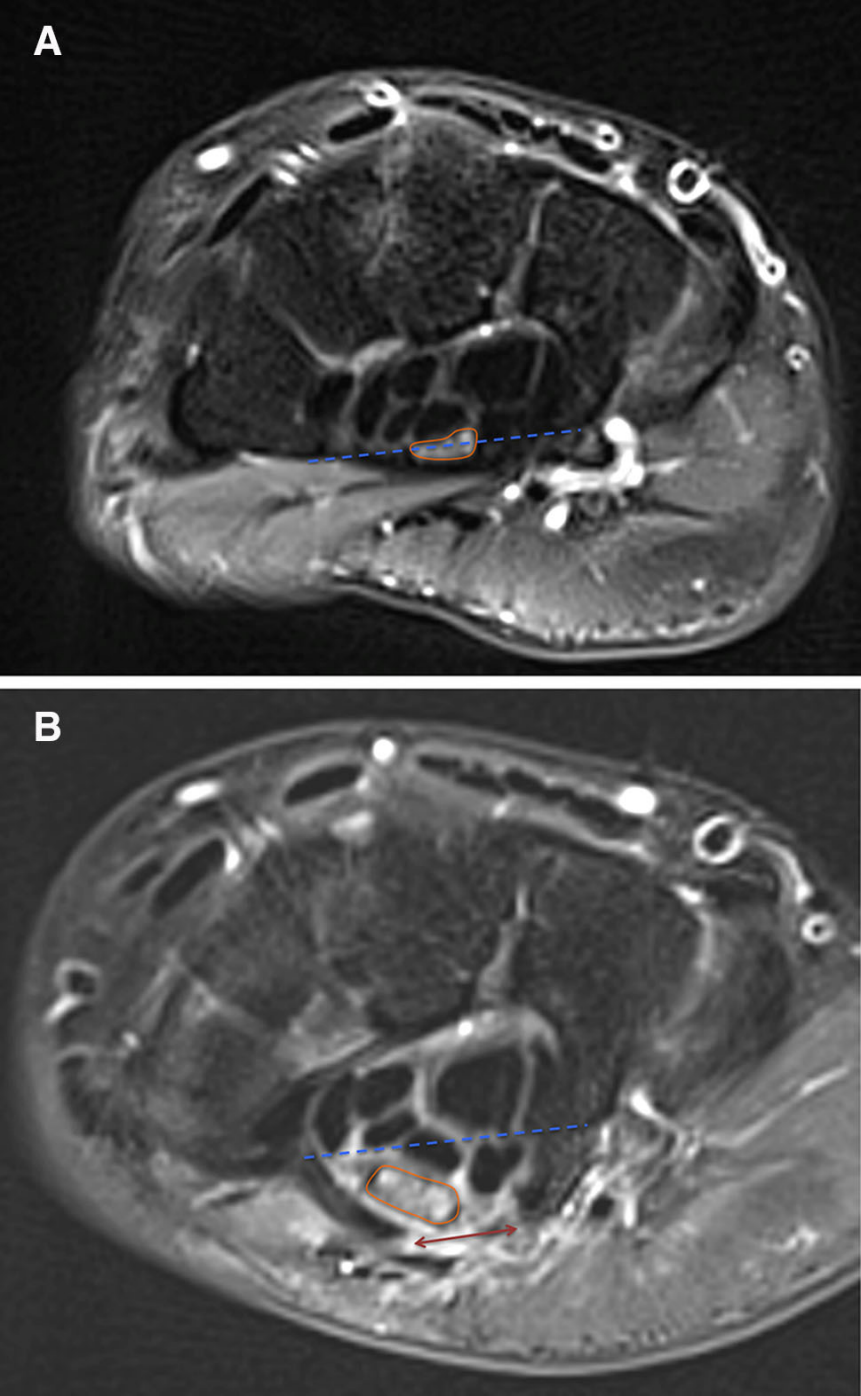
Magnetic resonance images before and after minimally invasive ultrasound-guided percutaneous carpal tunnel release surgery. Magnetic resonance imaging using axial T2-weighted fat saturation sequences at the level of the hook of hamate. **A** Pre-operative. The median nerve is compressed by the TCL. The median nerve crosses the line connecting the hook of hamate to the ridge of the trapezium (scored as intermediary position). **B** 3 months after procedure. Gap in the TCL (*double arrow*). The median nerve is below the line connecting the hook of hamate to the ridge of the trapezium (scored as more superficial). The median nerve became rounder and larger. The *continuous line* surrounds the cross-sectional area of the median nerve. The *dotted line* connects the hook of hamate to the ridge of the trapezium

Nerve position changed in 79% of patients (95% confidence interval: [66.8–91.2%]; Table 1). An example of MRI images before and after successful PCTR is presented in Fig. 5. After PCTR, the median nerve became rounder and larger at the hamate level, and the nerve position changed from being at the same level as the line joining the hook of hamate and the ridge of the trapezium to being below it.

**Table 1:** Median nerve position 1 month after percutaneous carpal tunnel release (*N* = 129)

Position before PCTR	Position after PCTR		
	Nerve above the line	Nerve crosses the line	Nerve under the line
Nerve above the line	12 (13.8%)	27 (31.0%)	48 (55.2%)
Nerve crosses the line	0	12 (30.8%)	27 (69.2%)
Nerve under the line	0	0	3 (100%)

Position of the nerve was compared to the line joining the hook of hamate to the ridge of the trapezium. Decompression was considered successful if nerve location went from a deep to a superficial position: nerve above the line (deepest), nerve crosses the line (intermediary); nerve under the line (most superficial). *PCTR* percutaneous carpal tunnel release

## Discussion

In this study, which included patients with moderate to severe carpal tunnel syndrome (baseline BCTQ symptom severity and functional status scores of 3.3 and 2.6, respectively), we performed the retrograde division of the transverse carpal ligament using an ultra-minimally invasive ultrasound-guided percutaneous procedure. Recent papers have described this technique [6, 9, 11, 14], but all performed by orthopedist or interventional rheumatologist with unknown years of experience with hand ultrasound; therefore, there have been questions about their generalizability with a large list of technical contraindications such as a distance between the median nerve and the ulnar artery less than 3 mm or anatomical variation of the median nerve [20, 21]. Rojo in his cadaveric study concluded that PCTR may be difficult for an orthopedist without previous experience in ultrasound-guided procedures and should develop his skills first with guided infiltration (8). In this first study performed by an interventional radiologist with more than ten years’ experience with hand ultrasound and US-guided percutaneous procedure, there were no technical limitations.

One-month and 6-month post-operative clinical results showed that mean BCTQ scores had improved significantly, both in the symptom severity and the functional status domains in all patients similar to that of OCTR and ECTR (2). Magnetic resonance imaging results 1 month after the procedure showed that the transverse carpal ligament was completely sectioned in 100% of patients. There were no intra or post-operative complications among the patients. The clinical and MRI results of this study show that ultrasound monitoring can be used successfully by an interventional radiologist to guide incision points and release paths and could be a good alternative to current minimally invasive methods.

Magnetic resonance imaging was performed 1 month after the procedure in order to confirm complete resection of the TCL. We found that at the 1-month time point, sectioning of the TCL was visible and that sectioning was complete along its length in all 129 patients. Change in nerve position compared to the line joining the hook of hamate to the ridge of the trapezium is a measure of decompression that can be assessed by MRI. More superficial placement indicates better decompression. In 79% of patients, the nerve position became more superficial after the procedure, and in 88% of patients, no paresthesia was reported at 6 months. The long-term significance of these data is unknown, but Campagna et al. [17] showed that insufficient change in nerve position was associated with carpal tunnel syndrome recurrence. We can therefore surmise that the incidence of recurrence, in our patient population would be low.

No nerve damage-related complications were identified on Month 1 MRIs or reported during clinical evaluations. These data, which were collected in a cohort that included 15 patients with a bifid median nerve, suggest that real-time continuous ultrasound imaging provided sufficient visual support to avoid nerve damage. Good clinical outcomes and no clinical indication of nerve damage have also been recorded in other studies that used ultrasound to guide the procedure [6, 9, 11, 14]. As our study was not designed to collect anatomical data about variations of the palmar cutaneous branch and thenar motor branches and was not sufficiently powered to enroll patients with a large range of anatomic variations [20–22], larger studies that include a wide range of anatomical presentations and that specifically document anatomical variations will be needed to determine complication rates in higher risk patients.

We found that our technique was truly minimally invasive as scar length ranged from 2.0 to 5.0 mm. Small incision points imply less infection and less potential for scar pain. Consistent with this observation, no pain or infections were reported. By contrast, in OCTR and ECTR studies, over 50% of patients report scar pain [23] and patients have cited concerns about scar pain as a reason for surgery cancellation [24]. Although the causes of scar pain are not well understood, we can hypothesize that a smaller incision will be associated with less superficial skin nerve damage and therefore less scar discomfort. In fact, in the comparative study by Nakamichi et al. [6], patients reported less scar sensitivity with a 4 mm incision from PCTR than with a 10–15 mm incision from mini-OCTR.

Short procedure duration times suggest less potential for infection and lower procedure cost. The mean PCTR procedure duration was 6 min. These data contrast with that reported by Lecoq et al. [14] who reported PCTR procedure times of 19 min. In this study, the operator was an interventional orthopedist or rheumatologist trained to ultrasound. However, the number of years of experience with hand ultrasound is unclear. We have to assume that, at this early stage of procedure development, differences in technique and training, particularly between rheumatologists, interventional radiologists, and surgeons, are likely to affect the duration of this procedure, which has been described by some as being technically demanding [11].

Lastly, this study is one of the first studies to perform PCTR in the radiology intervention room [14]. The absence of sepsis and complications supports the apparent feasibility and safety of performing this procedure outside of the operating room.

### Limitations

In our study, efficacy was measured based on TCL sectioning, nerve decompression, and patient-reported symptoms. Other measures such as grip strength and atrophy were not measured. This represents a limitation in our interpretation of efficacy as in some PCTR studies, significant clinical improvement was nonetheless accompanied by >30% of patients having atrophy in their hand and/or below-average grip strength [9]. As these studies did not use MRI to document complete sectioning and nerve decompression, it is unclear whether these data reflect only partial surgical success.

Furthermore, this is a single operator cohort study that needs to be confirmed by a randomized multicenter controlled trial evaluating safety and efficacy of PCTR versus ECTR or OCTR.

Additional studies would be needed to understand the relationship between the degree of sectioning and decompression and symptoms such as grip strength.

## Conclusions

In our study, we show that ultrasound-guided PCTR was used successfully to section the transverse carpal ligament, decompress the median nerve, and improve self-reported symptoms. Magnetic resonance imaging results showed that outcomes were similar to those reported after OCTR with a complete sectioning of the ligament and successful decompression of the nerve.

## References

[1] Scholten RJ, Mink van der Molen A, Uitdehaag BM, Bouter LM, de Vet HC. Surgical treatment options for carpal tunnel syndrome. Cochrane Database Syst Rev. 2007;(4):CD003905. 10.1002/14651858.CD003905.pub3 PMC6823225 17943805

[2] Kohanzadeh S, Herrera FA, Dobke M (2012). Outcomes of open and endoscopic carpal tunnel release: a meta-analysis. Hand (N Y).

[3] Sayegh ET, Strauch RJ (2015). Open versus endoscopic carpal tunnel release: a meta-analysis of randomized controlled trials. Clin Orthop Relat Res.

[4] Vasiliadis HS, Georgoulas P, Shrier I, Salanti G, Scholten RJ (2014). Endoscopic release for carpal tunnel syndrome. Cochrane Database Syst Rev.

[5] Tagliafico A, Pugliese F, Bianchi S (2008). High-resolution sonography of the palmar cutaneous branch of the median nerve. AJR Am J Roentgenol.

[6] Nakamichi K, Tachibana S, Yamamoto S, Ida M (2010). Percutaneous carpal tunnel release compared with mini-open release using ultrasonographic guidance for both techniques. J Hand Surg Am.

[7] Lecoq B, Hanouz N, Vielpeau C, Marcelli C (2011). Ultrasound-guided percutaneous surgery for carpal tunnel syndrome: a cadaver study. Joint Bone Spine.

[8] Rojo-Manaute JM, Capa-Grasa A, Rodriguez-Maruri GE, Moran LM, Martinez MV, Martin JV (2013). Ultra-minimally invasive sonographically guided carpal tunnel release: anatomic study of a new technique. J Ultrasound Med.

[9] McShane JM, Slaff S, Gold JE, Nazarian LN (2012). Sonographically guided percutaneous needle release of the carpal tunnel for treatment of carpal tunnel syndrome: preliminary report. J Ultrasound Med.

[10] Markison RE (2013). Percutaneous ultrasound-guided MANOS carpal tunnel release technique. Hand (N Y).

[11] Chern TC, Kuo LC, Shao CJ, Wu TT, Wu KC, Jou IM (2015). Ultrasonographically guided percutaneous carpal tunnel release: early clinical experiences and outcomes. Arthroscopy.

[12] Rowe NM, Michaels J 5th, Soltanian H, Dobryansky M, Peimer CA, Gurtner GC. Sonographically guided percutaneous carpal tunnel release: an anatomic and cadaveric study. Ann Plast Surg. 2005;55:52–6; discussion 56. 10.1097/01.sap.0000168281.77528.02 15985791

[13] Chern TC, Wu KC, Huang LW (2014). A cadaveric and preliminary clinical study of ultrasonographically assisted percutaneous carpal tunnel release. Ultrasound Med Biol.

[14] Lecoq B, Hanouz N, Morello R (2015). Ultrasound-assisted surgical release of carpal tunnel syndrome: results of a pilot open-label uncontrolled trial conducted outside the operating theatre. Joint Bone Spine.

[15] Cudlip SA, Howe FA, Clifton A, Schwartz MS, Bell BA (2002). Magnetic resonance neurography studies of the median nerve before and after carpal tunnel decompression. J Neurosurg.

[16] Momose T, Uchiyama S, Kobayashi S, Nakagawa H, Kato H (2014). Structural changes of the carpal tunnel, median nerve and flexor tendons in MRI before and after endoscopic carpal tunnel release. Hand Surg.

[17] Campagna R, Pessis E, Feydy A (2009). MRI assessment of recurrent carpal tunnel syndrome after open surgical release of the median nerve. AJR Am J Roentgenol.

[18] Levine DW, Simmons BP, Koris MJ (1993). A self-administered questionnaire for the assessment of severity of symptoms and functional status in carpal tunnel syndrome. J Bone Joint Surg Am.

[19] Leite JC, Jerosch-Herold C, Song F (2006). A systematic review of the psychometric properties of the Boston Carpal Tunnel Questionnaire. BMC Musculoskelet Disord.

[20] Hurwitz PJ (1996). Variations in the course of the thenar motor branch of the median nerve. J Hand Surg Br.

[21] Granata G, Caliandro P, Pazzaglia C (2011). Prevalence of bifid median nerve at wrist assessed through ultrasound. Neurol Sci.

[22] Mitchell R, Chesney A, Seal S, McKnight L, Thoma A (2009). Anatomical variations of the carpal tunnel structures. Can J Plast Surg.

[23] Atroshi I, Larsson GU, Ornstein E, Hofer M, Johnsson R, Ranstam J (2006). Outcomes of endoscopic surgery compared with open surgery for carpal tunnel syndrome among employed patients: randomised controlled trial. BMJ.

[24] Gong HS, Baek GH, Oh JH, Lee YH, Jeon SH, Chung MS (2009). Factors affecting willingness to undergo carpal tunnel release. J Bone Joint Surg Am.

